# Increased IGF‐1 and DNA Synthesis in Patients With Activation‐Induced Cytidine Deaminase Deficiency

**DOI:** 10.1155/jimr/9495312

**Published:** 2026-04-15

**Authors:** Gursev Dhaunsi, Waleed Al-Herz

**Affiliations:** ^1^ Department of Pediatrics, College of Medicine, Kuwait University, Jabriya, Kuwait, kuniv.edu

**Keywords:** activation-induced cytidine deaminase, DNA synthesis, IGF-1, inborn errors of immunity, lipid peroxidation

## Abstract

**Objective:**

To characterize patients with activation‐induced cytidine deaminase (AICDA) deficiency, a rare autosomal recessive inborn error of immunity (IEI), and examine the role of insulin‐like growth factor‐1 (IGF‐1) and cellular oxidative stress in the pathogenesis.

**Methods:**

Patients data was retrieved from the Kuwait National Primary Immunodeficiency Registry. Plasma IGF‐1 levels were tested by ELISA assay, while bromodeoxyuridine (BrdU) incorporation in peripheral blood mononuclear cells (PBMCs) was used as an index of DNA synthesis. IGF‐1 receptor (IGF‐1R) protein was measured by immunoblot analysis while PBMC NADPH oxidase activity and plasma malondialdehyde (MDA) levels were measured by colorimetric assays.

**Results:**

Nine patients with AICDA deficiency (6 with c.254G >A and 3 with c.169G >A variants) were included in this study. All patients suffered from bacterial infections, while four had lymphoproliferation. Plasma IGF‐1 and lipid peroxide (MDA) levels were significantly higher in AICDA‐deficient patients when compared with controls (*p*‐values <0.05 and <0.05, respectively). PBMCs isolated from AICDA deficient patients had significantly more IGF‐1‐stimulated BrdU incorporation than controls (*p*  < 0.01), while IGF‐1R levels were not statistically different. There was no statistical difference in plasma levels of IGF‐1 and DNA synthesis between patients with and without lymphoproliferation. NADPH oxidase activity in PBMC of AICDA patients and controls was not statistically different.

**Conclusion:**

We documented a novel finding of increased plasma levels of IGF‐1 in AICDA deficient patients. This may help in the future in better understanding the pathogenesis of the disease and possible use of targeted therapy or precision medicine.

## 1. Introduction

Activation‐induced cytidine deaminase (AICDA) enzyme selectively changes cytosine (C) residues into uracil (U), leading to the introduction of U:G mismatches on the single‐stranded DNA of transcribed switch (S) and variable regions of the Ig [1]. Defects in the AICDA gene result in an inborn error of immunity (IEI) that is characterized by failure of immunoglobulin (Ig) isotype class switch recombination (CSR) and somatic hypermutation in B lymphocytes [2]. Deficiency of AICDA is the most common autosomal recessive disorder among immunological diseases termed hyper IgM syndromes [3], where a defect in CSR initiation leads to the disease phenotype, and patients have high serum IgM and low or undetectable levels of IgG, IgA, and IgE compared to healthy subjects [4, 5]. The clinical phenotype of patients with defects in AICDA includes lymph node hyperplasia, and lymphadenopathy is a hallmark of the disease [6]. The enlargement of lymph nodes is so remarkable that patients usually undergo multiple biopsies [7]. AICDA‐deficient patients also suffer from bacterial infections affecting mostly the upper respiratory and the gastrointestinal tracts and they may also have autoimmune manifestations [8].

Hyperplastic activity in lymph nodes is a key feature of patients with AICDA deficiency [6], and it is well established that cellular proliferation during hyperplasia in various tissues involves activation of mitogen‐activated protein kinases, growth‐promoting peptides, and their receptors. It is intriguing to speculate if lymphadenopathy in AICDA patients is accompanied by growth factor‐mediated signaling and downstream effects. Growth factors such as insulin‐like growth factor‐1 (IGF‐1) have a crucial role in human development, growth, and function of different organs and tissues during different stages of life [9]. IGF‐1 is a peptide produced mainly by the liver; however, physiological production of IGF‐I also occurs in other tissues, such as growth plate and bone, with different autocrine and paracrine functions [10]. Serum levels of IGF‐1 vary with age with low levels at birth, highest levels during puberty, and a steady decrease in levels with aging [11]. IGF‐1 controls bone growth, cellular differentiation, and various metabolic functions, and its actions are dependent upon the availability and interactions of free IGFs with their receptors. Levels of free IGFs are regulated by the rate of IGF production, its clearance, and binding to the IGF‐binding proteins [12]. Abnormal serum levels of IGF‐1 were reported with malnutrition, catabolic states, and some chronic illnesses including inflammatory conditions [13]. Both IGF‐1 and its receptor (IGF‐1R) have been shown to be affected by the cellular oxidative stress due to overproduction of free radical species or impaired antioxidant defense in several diseases ranging from cardiovascular diseases to different types of malignancies [14]. Several studies on both humans and mice have shown that increased levels of IGF‐1 cause B‐cell lymphopoiesis, further underscoring the importance of IGF‐1 in the regulation of various cellular functions under both physiological and pathological states [15]. IGF‐1 and its receptor have also been reported to play a crucial role in blood disorders and immunological responses, including promotion of development and cytotoxic activity of human natural killer cells [16].

Human inborn errors of immunity (IEI) provide unique grounds to study the immune system and its regulatory factors and are thus an exceptional virtue from both physiological and pathological perspectives. Additionally, there is a significant knowledge gap about the cause of different manifestations in IEI. In this project we speculated that lymphoproliferation in patients with AICDA defect involves enhanced mitogenic activities leading to lymphoproliferation and examined whether IGF‐1 has any role in the pathogenesis of AICDA defects either through its increased production or upregulation of its receptor. Better understanding of different pathophysiologic changes in IEI may raise the possibility of using targeted therapy to control different disease manifestations.

## 2. Methodology

### 2.1. Subjects’ Recruitment and Sample Collection

All AICDA‐deficient patients (6 males and 3 females) who are registered in the Kuwait National Primary Immunodeficiency Registry were included in the study. Six males and three females aged matched healthy control subjects without any growth‐related and/or immunological conditions were also included. The study was approved by the Research and Ethics Committee of the Ministry of Health in Kuwait and by the Kuwait University Health Sciences Center Ethical Committee, in accordance with the Declaration of Helsinki and written informed consent was signed by all subjects or their legal guardians. Venous blood samples (8 mL) were collected from all subjects for isolation of genomic DNA, plasma, and peripheral blood mononuclear cells (PBMC). Plasma and DNA samples were stored until further testing by biochemical and molecular assays. Participants did not have evidence of infection at the time of blood sampling. AICDA‐deficient patients were on monthly intravenous immunoglobulins, while the control subjects were not on medications.

### 2.2. PBMC Cultures and BrdU Incorporation

PBMCs were isolated by the density gradient centrifugation technique using Ficoll‐Paque. Isolated PBMCs were washed twice with RPMI 1640 medium, and then cultures were set up in 96‐well culture plates for experiments to examine DNA synthesis by BrdU incorporation assay. To study the mitogenic action of IGF‐1, cell cultures were incubated in culture medium containing a low concentration of fetal bovine serum (1% FBS) for 24 h before treatment with 100 ng/mL of IGF‐1 (Abcam, Cambridge, United Kingdom). BrdU incorporation into PBMC was measured as an index of DNA synthesis using ELSA kits from Calbiochem (CA, USA).

### 2.3. IGF‐1R Detection Using Western Blot Analysis

Cell homogenates of PBMC were lysed in buffer (pH 7.6) containing 50 mM Tris‐base, 5 mM EGTA, 150 mM NaCl, 1% Triton 100, 2 mM Na_3_VO_4_, 50 mM NAF, 1 mM PMSF, 20 μM phenylarsine, 10 mM sodium molybdate, 10 μg/mL leupeptin, and 8 μg/mL aprotinin. Lysates containing equal amounts of protein were subjected to SDS–polyacrylamide gel electrophoresis (SDS–PAGE) and then transferred onto a nitrocellulose membrane. Membranes were then incubated with human‐specific anti‐IGF‐1R antibodies (ab131476) obtained from Abcam (Cambridge, United Kingdom), and IGF‐1R protein was detected using goat anti‐mouse HRP‐labeled polyclonal secondary antibodies (ab205719) procured from Abcam (Cambridge, United Kingdom). Bands were detected with SuperSignal chemiluminescent substrate. To ensure equal loading of proteins, *β*‐actin levels were detected using a primary rabbit anti‐human *β*‐actin antibody. Images were analyzed and quantified by densitometry.

### 2.4. IGF‐1 Assay

Total IGF‐1 concentrations in plasma samples were measured using commercially available ELISA kits from Assay Design Inc (Ann Arbor, MI, USA).

### 2.5. Lipid Peroxidation and NADPH Oxidase Activity Assays

Malondialdehyde (MDA) levels were measured in cell homogenates as an index of lipid peroxide production. A Calbiochem kit was used to assay MDA levels spectrophotometrically using a plate reader with a 500 nm filter. NOX activity was measured in cell homogenates using a method described earlier by Rajagopalan et al. [17]. Briefly, a known amount of cell homogenate was added to a reaction mixture that contained 50 mM phosphate buffer, EDTA (0.01 mM), and 25 µM lucigenin. The reaction was started by the addition of 100 µM NADPH, and the chemiluminescence was recorded at 495 nm. NOX activity was calculated as relative light units (RLUs) emitted/mg protein/min.

### 2.6. Statistical Analysis

Mean ± Standard Deviation (SD) values were calculated, and data was analyzed using student’s *t*‐ test for significance of variance between different groups of the study. Multiple comparisons were carried out by one‐way analysis of variance using IBM SPSS Statistics 29.0 software (New York, United States), and the statistical significance level was set at *α* = 0.05.

## 3. Results

### 3.1. Demographic and Clinical Features of AICDADeficient Patients in Kuwait

Table 1 shows the demographic, clinical, and molecular details of nine patients with pathogenic variants in the AICDA gene. All patients presented with bacterial infections, while only 4 patients (44%), all males, showed lymphoproliferation. 67% of patients with c.254G >A variant showed lymphoproliferation, while none of the patients with c.169G>A variant showed lymphoproliferation.

**Table 1:** Demographic, clinical characteristics and genetic variants of patients with AICDA gene defect.

Patient no.	Patient code	Age (years)	Sex	Clinical manifestations			AICDA mutation
				Infections	Lymphoproliferation	Autoimmunity	
1	B9	33	M	Sinopulmonary	+	ITP/SLE	c.254G >A
2	B11	26	M	Sinopulmonary	+	—	c.254G >A
3	B31	14	M	Sinopulmonary	—	—	c.254G >A
4	B22	23	M	Sinopulmonary	+	—	c.254G >A
5	B7	20	F	Sinopulmonary	—	—	c.254G >A
6	B10	20	M	Sinopulmonary	+	—	c.254G >A
7	B30	19	M	Sinopulmonary Bacteremia	—	Psoriasis	c.169G >A
8	B43	7	F	Sinopulmonary	—	—	c.169G >A
9	B23	11	F	Bacteremia	—	—	c.169G >A

### 3.2. Increased Plasma IGF‐1 Levels in AICDADeficient Patients

**Figure 1:**
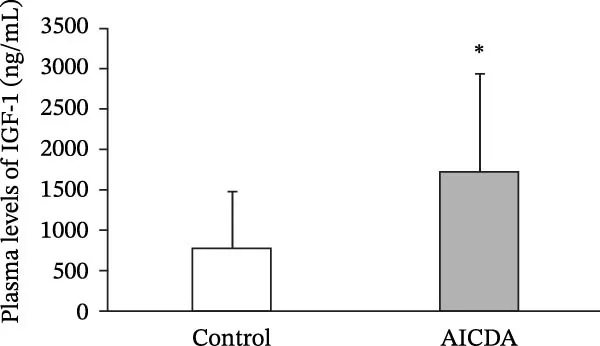
Plasma IGF‐1 levels are higher in AICDA deficient patients (*n* = 9) compared to healthy control subjects (*n* = 9) ( ^∗^ *p*  < 0.05). Data shown are mean ± SD of values obtained from single assays carried out in duplicate on nine control samples and nine patient samples.

AICDA‐deficient patients showed significantly (*p*  < 0.05) higher levels of plasma IGF‐1 (1713 ± 1254 ng/mL) as compared to healthy controls (766 ± 706 ng/mL) as shown in Figure 1, suggesting a possible mitogenic role for IGF‐1 in AICDA deficient subjects. Plasma levels of IGF‐1 were higher, but not statistically significant (*p* = 0.49) in male AICDA‐deficient patients (1935 ± 1429 ng/mL) as compared to the female ones (1268 ± 861 ng/mL). There was no statistical difference (*p*  = 0.33) in plasma levels of IGF‐1 between patients with (1229 ± 607 ng/mL) and without lymphoproliferation (2100 ± 1565 ng/mL).

### 3.3. Increased DNA Synthesis (as Determined by BrdU Incorporation) in AICDADeficient Patients

**Figure 2:**
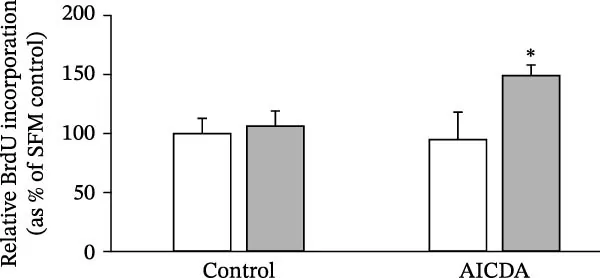
BrdU incorporation in PBMC of AICDA deficient patients (*n* = 9) is higher ( ^∗^ *p*  < 0.01) compared to healthy control subjects (*n* = 9) following cultures in the absence (open bars) or presence (dark bars) of IGF‐1 (100 ng/mL) for 24 h. Data shown are mean ± SD of values obtained from single assays carried out in duplicate on nine control samples and nine patient samples.

Figure 2 shows that stimulation of serum‐starved cells with IGF‐1 (100 ng/mL) resulted in a significant (*p*  < 0.01) increase in DNA synthesis in AICDA patients (149.0 ± 9.6) when compared with control subjects cells (105.0 ± 13.8), strongly indicating a role for IGF‐1 in the pathogenesis of AICDA. There was no statistical difference in DNA synthesis between patients with and without lymphoproliferation.

### 3.4. Comparable IGF‐1R Content Both in AICDADeficient Patients and Healthy Controls

**Figure 3:**
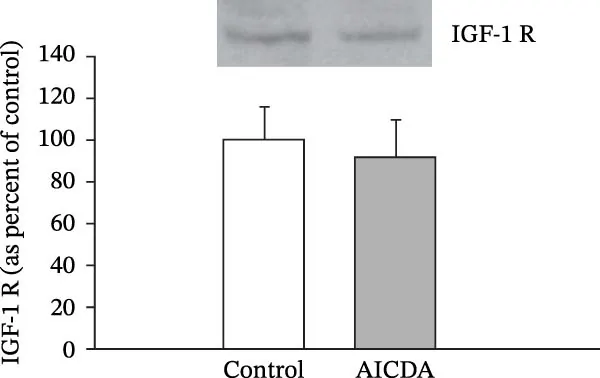
Immunoprecipitation/Western blot detection of IGF‐1 receptor levels in PBMC isolated from AICDA deficient patients (*n* = 9) shows no statistical difference compared to healthy control subjects (*n* = 9). Protein bands shown are from a representative immunoblot. Data presented as percent of controls are the mean ± SD of densitometry values.

Immunoprecipitation/Western blot analysis revealed that IGF‐1R protein content (shown as percent of control in Figure 3 with a representative blot and Figure S1) in PBMC of AICDA patients was not statistically different (*p* = 0.76) when compared with that in PBMC of control subjects.

### 3.5. High Lipid Peroxidation in AICDADeficient Patients

**Figure 4:**
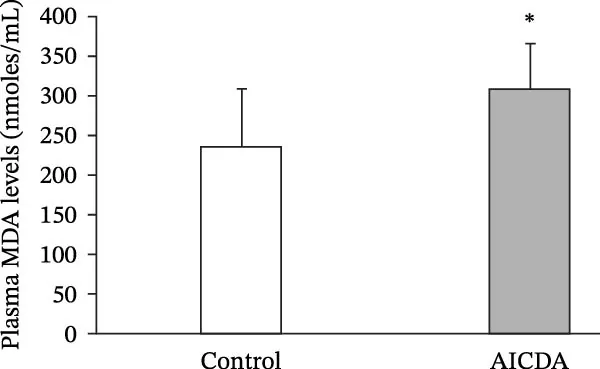
Plasma levels malondialdehyde (ng/mL) in AICDA deficient patients (*n* = 9) is higher compared to healthy control subjects (*n* = 9) ( ^∗^ *p*  < 0.05). Data shown are mean ± SD of values obtained from single assays carried out in duplicate on nine control samples and nine patient samples.

**Figure 5:**
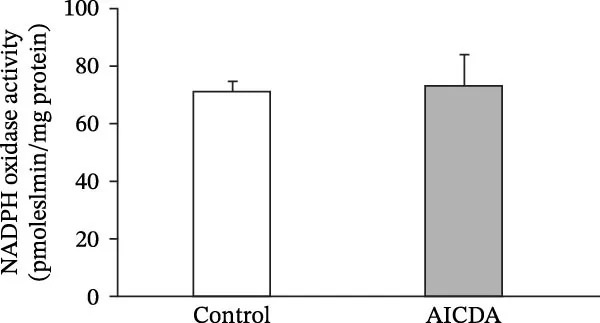
NADPH oxidase activity (pmoles/min/mg protein) in PBMC isolated from AICDA deficient patients (*n* = 9) shows no statistical difference compared to healthy controls. Data shown are mean ± SD of values obtained from enzymes assays carried out in duplicate on nine control samples and nine patient samples.

Figure 4 shows that plasma levels of MDA levels (an index of lipid peroxidation/oxidative stress) were significantly (*p*  < 0.05) higher in AICDA patients compared to the levels in plasma of control subjects, demonstrating some evidence of cellular oxidative stress associated with AICDA deficiency. To examine if the observed increase in cellular oxidative stress in AICDA‐deficient patients was due to increased superoxide anion production, NOX activity was assayed in PBMC of both groups. Figure 5 shows that the NOX activity was not statistically different (*p* = 0.61) in the AICDA gene defect, as both patients and controls showed similar levels of NOX enzyme activity in PBMC.

## 4. Discussion

AICDA deficiency is an IEI characterized by a complete lack of immunoglobulin CSR and somatic hypermutation [2]. The clinical phenotype in our cohort is in accordance with previous reports [4–7], except for lymphoproliferation, which affected <50% in our patients compared to nearly 75% in one study [6].

In the current study, we report a novel finding that patients with an AICDA gene defect have significantly elevated plasma IGF‐1 levels, and their PBMC have a markedly enhanced mitogenic response to IGF‐1. Although the exact mechanism causing the observed increase in plasma IGF‐1 levels in AICDA‐deficient patients remains unknown, it can only be speculated that immunological responses due to AICDA gene defect might trigger some cellular and metabolic events in the liver, a major organ responsible for IGF‐1 production. Elevated plasma IGF‐1 levels have been observed in certain inflammatory conditions such as infectious diseases, cancer, and autoimmune disorders that involve overproduction of reactive oxygen species and cellular oxidative stress [18, 19]. Several pathological conditions, including infections, atherogenesis, malignancies, and lymphadenopathy are known to be associated with increased cellular oxidative stress. Lipid peroxidation has been widely studied as an indicator of cellular oxidative stress as lipid moieties are easy targets for reactive oxygen species due to the presence of carbon–carbon double bonds, particularly in polyunsaturated fatty acids [14, 18, 20]. AICDA‐deficient patients in this study showed some signs of oxidative stress as levels of MDA, an indicator of lipid peroxidation, were markedly elevated in their plasma. The observed increase in the oxidative stress in the plasma of AICDA‐deficient patients could either be due to the systemic effect of immunological mediators or some specific effect on circulating blood cells. Further, unaltered NADPH oxidase activity indicates that NOX‐mediated superoxide anion production could not be responsible for the observed oxidative stress in PBMC of AICDA‐deficient patients. Rather, antioxidant system, enzymatic or non‐enzymatic, might be impaired. There are reports in the literature that suggest mitophagy and a related cascade of metabolic events due to AICDA dysfunction that cause oxidative stress [21]. In view of the common feature of lymphoproliferation in AICDA‐deficient patients, it can be speculated that abnormally elevated IGF‐1 levels might be promoting mitogenic signals to induce cellular proliferation in lymphoid tissues. Though IGF‐1 levels were not markedly different between AICDA‐deficient patients with or without lymphoproliferation, probably due to the low number of patients studied, these findings do not rule out the importance of IGF‐1 and some other growth factors in the overall pathogenesis of AICDA deficiency.

This study further revealed that PBMCs of AICDA‐deficient patients showed a marked increase in DNA synthesis in response to IGF‐1. Though IGF‐1‐induced BrdU incorporation in PBMC of AICDA‐deficient patients with lymphadenopathy was not markedly different from those without lymphoproliferation, probably due to the low number of patients included, this finding specific to PBMC do not rule out participation of IGF‐1 in overall pathogenic events of AICDA deficiency. IGF‐1 and other growth factors carry out mitogenic actions through their specific receptors. Protein expression of IGF‐1R in PBMC was not affected by AICDA deficiency, suggesting that enhanced IGF‐1‐induced DNA synthesis in AICDA‐deficient patients might have occurred through transactivation of other homologous receptors such as EGFR or insulin receptors as reported in other studies [22, 23]. Findings of this study, though limited to PBMC, strongly suggest that IGF‐1 and other growth factors are known to play a significant role in cellular proliferation under various pathophysiological conditions such as malignancies and vascular diseases, might contribute toward pathogenic events, such as lymphoproliferation in AICDA deficiency. Our experiments show that studying patients with IEI can shed light on how the human immune system is regulated. Such studies deepen our understanding of immune mechanisms by revealing previously unrecognized functions of various genes and cells involved in host defense.

In conclusion, this study establishes a possible role for IGF‐1 in the pathogenesis of AICDA deficiency. The limitation in our study is the low number of included patients due to the rarity of this specific IEI. This should encourage future collaborative multicenter detailed studies for better understanding of the exact role of growth factors in the pathophysiology of AICDA deficiency, which may open the door for the use of targeted therapy and precision medicine.

## Funding

This study was supported by a Research Grant (MK 01/22) from Research Sector, Kuwait University.

## Ethics Statement

The study was approved by the Research and Ethics Committee of the Ministry of Health in Kuwait and by the Kuwait University Health Sciences Center Ethical Committee, in accordance with the Declaration of Helsinki, and written informed consent was signed by all subjects or their legal guardians.

## Conflicts of Interest

The authors declare no conflicts of interest.

## Supporting Information

Additional supporting information can be found online in the Supporting Information section.

## Supporting information


**Supporting Information** Immunoprecipitation and Western blots for IGF‐1 receptor analysis. C1–C9 are controls samples and P1–P9 are patients samples. Blots 1 and 2 were done parallel to each other with similar conditions, and blots 3 and 4 were done parallel to each other with similar conditions. Blot 5 shows samples C3 and P3 were run as repeat with molecular weight markers.

## Data Availability

The data underlying this article will be shared on reasonable request to the corresponding author.
