# Supplementary material for: Increased IGF‐1 and DNA Synthesis in Patients With Activation‐Induced Cytidine Deaminase Deficiency
Source: J Immunol Res. 2026 Apr 15;2026:9495312. doi: 10.1155/jimr/9495312 (PMC13581056; doi:10.1155/jimr/9495312)
Supplement: Supplementary file 1 [file JIMR-2026-9495312-s001.docx]

**Supplementary Figure 1:**

Immunoprecipitation and Western blots for IGF-1 receptor analysis. C1-C9 are controls samples and P1-P9 are patients samples. Blots 1 and 2 were done parallel to each other with similar conditions, and blots 3 and 4 were done parallel to each other with similar conditions. Blot 5 shows samples C3 and P3 were run as repeat with molecular weight markers.

Blot 1	Blot 2

Blot 3	Blot 4

Blot 5
